# Comment on Oehler et al. Outcome and Midterm Survival after Heart Transplantation Is Independent from Donor Length of Stay in the Intensive Care Unit. *Life* 2022, *12*, 1053

**DOI:** 10.3390/life13071443

**Published:** 2023-06-26

**Authors:** Myat Soe Thet, Alessandra Verzelloni Sef, Nicholas J. Lees, Davorin Sef

**Affiliations:** 1 Department of Surgery and Cancer, Faculty of Medicine, Imperial College London & Imperial College Healthcare NHS Trust, London W2 1NY, UK; 2 Department of Anaesthesia and Critical Care, Harefield Hospital, Royal Brompton and Harefield Hospitals, London UB9 6JH, UK; 3 Royal Brompton and Harefield Hospitals, Harefield Hospital, London UB9 6JH, UK

Oehler et al. described an interesting finding, stating that length of stay (LOS) of the donors in the intensive care unit (ICU) did not have an impact on the outcomes and survival of recipients up to 5 years after heart transplantation (HTx) [1]. Since donor LOS is not a commonly reported characteristic in the literature, the impact of prolonged donor ICU stay on survival after HTx has not been well examined. A few studies attempted to identify the correlation of donor ICU stay with post-transplant outcomes [2,3]. However, the authors extended their discussion by including studies related to post-cardiac surgery survival, although this is a completely different setting from the organ donation and post-transplant outcomes.

One of the limitations of the study, which the authors have not acknowledged, is that it includes solely donors with donation after brain death (DBD), obviously due to ethical reasons. Recently, donation after circulatory death (DCD) has been developed to expand the donor pool, yielding comparable outcomes to DBD, resulting in an increase in HTx volume by almost 50% [4]. Since DCD solid organ donors tend to have longer donor LOS in the ICU compared to DBD donors, it would be interesting to see whether the findings from this study can apply to DCD heart donors, given the projected increase in DCD HTx in the coming years [5,6]. Furthermore, the authors have not described their institutional donor organ procurement and HTx surgical techniques, which makes it more difficult to draw conclusions from their study and reported results [7,8,9].

Interestingly, the authors more commonly observed mild hypernatremia among the donors with medium LOS. There is an ongoing debate regarding the effect of donor serum sodium levels on the outcomes of solid organ transplants, and it is not an exception in HTx [10,11,12,13]. Recently, the same group already reported a retrospective study examining the effect of donor hypernatremia over a similar period with a large overlapping patient population [14]. They observed worse short-term survival among patients with hypernatremia. Although outcomes in the current study do not seem to be affected by hypernatremia, this observation highlights a confounding factor of hypernatremia [1]. Additionally, the authors reported lower haemoglobin levels in the subgroup with the longest LOS in the ICU as compared with the outher two subgroups (9 g/dL vs. 11 g/dL and 10 g/dL). However, they did not analyse or discuss the reasons behind this. Furthermore, the subgroup with the longest LOS in the ICU in this cohort experienced around twice less cardiopulmonary resuscitation prior to organ donation compared to the other two subgroups (15.9% vs. 29.3% and 38.8%). Surprisingly, the same subgroup had less infection/sepsis and pulmonary hypertension compared to the short LOS subgroup (19.7% vs. 29.7%, 5.8% vs. 12%, respectively), and we believe these are all important confounding factors to consider regarding their conclusions, as these three subgroups of donors are not similar in terms of clinical characteristics. Furthermore, it would be interesting to know how many recipients among these groups were bridged to HTx with mechanical circulatory support or had prior sternotomies, as several studies have described a potentially negative effect on post-transplant survival [15,16,17]. It is also well known that prior sternotomies are associated with increased morbidity and mortality of recipients after HTx due to several factors, such as higher intraoperative blood use, postoperative LOS in the ICU and hospital, and re-exploration for bleeding [17]. Lastly, since donor brain stem death is a significant contributor to donor heart dysfunction and primary graft dysfunction (PGD), it would be important to analyse whether donors with a prolonged LOS in the ICU were associated with higher rates of PGD [18]. A more comprehensive understanding of this problem could help to identify important cardioprotective strategies to improve the number and quality of donor hearts, and we hope that future studies will provide better answers to some of these questions.

The evaluation of heart donors is based on the assessment of many factors, and three main international transplant risk scoring systems have been developed to aid evaluations, including the United Network for Organ Sharing (UNOS) score, the Eurotransplant donor score, and the RADIAL score [19,20,21]. Importantly, Murana et al. recently compared these scoring systems in their study of 461 consecutive adult HTx recipients and found that, among the donor-related factors, a high noradrenaline support negatively influenced early post-transplant outcomes and survival, whereas an ischaemic time >240 min influenced early graft failure occurrence [2]. However, as the authors observed that the above-mentioned scoring systems mostly failed to demonstrate a significant correlation with outcomes in their population, they concluded that local donor factors based on a single-centre experience should be considered during graft selection [2].

Nevertheless, Oehler et al. have paved the way for further research to examine the effect of donor LOS on the outcomes of HTx. Adequately powered prospective studies in the future are needed to better understand this ongoing dilemma.

## Data Availability

The data presented in this study are available upon request from the corresponding authors.
